# Distribution and clinical relevance of phospholipids in hepatocellular carcinoma

**DOI:** 10.1007/s12072-020-10056-8

**Published:** 2020-06-05

**Authors:** Zhirong Liu, Zhen Zhang, Hao Mei, Jinghe Mao, Xinchun Zhou

**Affiliations:** 1 Department of Biochemistry, Shanxi Medical University, Shanxi Province, Taiyuan, 030001 China; 2 Department of Computer Sciences, University of Mississippi Medical Center, Jackson, MS 39216 USA; 3 Department of Biology, Tougaloo College, Tougaloo, MS 39174 USA; 4 Department of Pathology, University of Mississippi Medical Center, Jackson, MS 39216 USA

**Keywords:** Hepatocellular carcinoma (HCC), Phospholipids (PL), Lipidomics, Gender, Race, Disparity, Biomarkers, Benign hepatic tissues (BHT), African American (AA), Caucasian American (CA)

## Abstract

**Background:**

Hepatocellular carcinoma (HCC) is the most common liver cancer and featured with prominent disparity in incidence and mortality rate between male and female. It remains unclear whether alterations of phospholipids (PL) in hepatic tissues contribute to the pathogenesis, progression, and disparity of HCC.

**Methods:**

Using electrospray ionization mass spectrometry (ESI–MS), PL profiles including 320 individual phospholipid species in 13 PL classes were determined in paired samples from HCC and adjacent benign hepatic tissues (BHT).

**Results:**

(1) Concentrations of PLs in most of individual species, in subgroups and in total were decreased in HCC than in BHT in all studied population; (2) the number of individual PL species significantly different between HCC and BHT, and the number of PLs in six subgroups and in total decreased in HCC were more in male population than in female population; (3) panels of PL parameters (more in male population than in female population) were identified as biomarkers in differentiation of HCC from BHT, and in the prediction of pathological grade and clinical stage of HCC with high sensitivity, specificity, and accuracy.

**Conclusion:**

It is concluded that alterations of PLs in hepatic tissues play important roles in pathogenesis, progression, and gender disparity of HCC.

**Electronic supplementary material:**

The online version of this article (10.1007/s12072-020-10056-8) contains supplementary material, which is available to authorized users.

## Introduction

Hepatocellular carcinoma (HCC) is a primary malignancy of liver with high morbidity and mortality worldwide [1]. HCC is known to be gender disparate, being more frequently occurred and aggressive in male than in female, accounting for 7.5% of total male cancers, and 3.4% of total female cancers, respectively [2]. The early detection and treatment can increase cure rate of HCC; however, the prognosis is poor for patients at advanced stage of HCC. To date, the molecular mechanisms underlying high morbidity, mortality, and gender disparity of HCC remain unclear.

Phospholipids (PLs) have diverse biological functions in cells. In addition to being building blocks in membranous structures, PLs also play important roles in cellular signaling and cell–cell interactions in tissues. Metabolic alterations of various PLs have been reported to link to cancer proliferation. However, differences in phospholipid profiles have been rarely studied between HCC and benign hepatic tissues (BHT), and not investigated between male and female populations.

Lipidomics is a powerful tool available for the analysis and characterization of lipids and their interacting moieties. Since the developments in mass spectrometry, advanced analytical methods are able to identify and quantify a large number of individual species, classes and subgroups of lipids, especially PLs at the same time [3]. Lipid profiling is now widely applied in the determination of lipid alterations between different pathological conditions in various cancers, such as renal clear cell carcinoma and prostate cancer [4, 5]. Regarding the HCC, lipid profiling has been mostly performed on body fluids in the identification of diagnostic biomarkers and in characterization of metabolic pathways for certain PLs. However, global PL profiles have not been performed on and compared between HCC and benign hepatic tissues (BHT). Cotte et al. performed electrospray ionization mass spectrometry (ESI–MS) for seven phospholipid classes on plasma samples from 45 HCC patients and 45 cirrhotic patients without HCC, suggesting that plasma phospholipid profiles related to HCC risk in liver cirrhotic patients, and some phospholipid species might serve as potential biomarkers in predicting HCC patient outcomes [6]. Another study reported that HCC is characterized by reduced ceramide, lower level of polyunsaturated (PUFA) phospholipids, and increased CE and SM [7]. Interestingly, a recent study determined phospholipid profiles in samples from both hepatic tissues and serum from 50 HCC patients using an untargeted lipidomics approach. The results showed that the most phospholipid species showed opposite tendency of changes between patients’ HCC tissues and sera, implying that lipid metabolisms are likely to be modulated in different manners in HCC tissues and peripheral circulations [8]. To date, comparison of PL profiles between HCC and adjacent benign hepatic tissues (BHT) from same patients has not been studied; and none of studies has linked the differences in hepatic PL profiles to gender disparity in incidence and mortality rate of HCC.

In the present study, we performed ESI–MS for 320 phospholipid species in 13 phospholipid classes on paired HCC and BHT tissues from 32 male and female HCC patients. Our aims are to determine differences in PL distribution between HCC and BHT, to reveal gender disparity in PL profiles and their clinic relevance between male and female populations, to identify PL biomarkers in the differentiation of HCC from BHT and in the prediction of progression of HCC, and to seek novel therapeutic targets in PL metabolisms for treatment of HCC.

## Materials and methods

### Patients and sample collection

This study was approved by the Institutional Review Board of the University of Mississippi Medical Center. The study cohort comprised 32 patients with clinical HCC. A total of 64 samples, including 32 HCC tissues and their accompanied BHT were serially collected by the Cooperative Human Tissue Network (CHTN). The written consents were obtained from patients prior to the donation of tissue samples. To insure quality and quantity of samples, each sample should be collected within 10 years and weighted > 50 mg. The diagnosis of each HCC and BHT sample was reconfirmed by the pathologists at sites patients were enrolled in. Histological features for BHT samples included normal liver tissues, inflammation/hepatitis, alcoholic fatty liver disease (AFLD), non-alcoholic fatty liver disease (NAFLD), and cirrhosis, but none of BHT samples contains liver cancer cells. Because the numbers in each of the categories with benign hepatic changes were limited, all non-cancer tissues adjacent to HCC were grouped as benign hepatic tissues (BHT). None of the identifiable information, such as patient’s name, date of birth, and contact information was provided. The geographic, clinical, and pathological information is listed in Table 1.

**Table 1:** Patient's geographic and clinic information

Features	*n* (%)
Age (*n*/mean ± SD)	32 (65.5 ± 13.8)
Race	32
CA	26 (81.25)
Others	6 (18.75)
Gender	32
Male	21 (65.63)
Female	11 (34.27)
Pathological grade	28^b^
Low grade (LG)	11
High grade (HG)	17
Clinical stage	28^a^
Early stage (ES)	20
Late stage (LS)	8
Margin	28^a^
Positive	3
Negative	25
Lymphovascular invasion	27^b^
Positive	12
Negative	15

^a^Lack of data on tumor grade, clinical stage and surgical margin in four cases

^b^l Lack of data on lymphovascular invasion in five cases

### Lipid extraction

Extraction of total lipids from HCC and BHT tissues was performed with chloroform and methanol, following a modified Bligh and Dyer protocol [9]. Briefly, 85–120 mg tissues was weighed and homogenized. To 0.8 part (volume) aqueous homogenized tissue, 1 part chloroform and 2 parts methanol were added and shaken well, followed by the addition of 1 part chloroform and 1 part water. The sample was shaken well, centrifuged at 3000 rpm for 5 min, and the lower layer was transferred to a glass vial. Then 1 part chloroform was added, the samples were shaken well, and centrifuged at 3000 rpm for 5 min, and the lower layer was transferred to the same glass vial; this process was repeated one more time. The lipid extract solvent collected in the glass vial was evaporated with liquid nitrogen, capped with a Teflon-lined cap, and transported to the KLRC Analytical Laboratory on dry ice for analysis.

### Protein extraction and quantification

Weighed fresh-frozen HCC or BHT tissues were minced and homogenized in RIPA buffer (50 mM Tris–Cl, pH 8.0, 150 mM NaCl, 1% NP-40, 0.5% sodium deoxycholate, 0.1% SDS) with protease inhibitor. The lysates were centrifuged, and the supernatants were collected and stored at − 80 °C. Protein concentration (mg/wet weight tissues, wwt) was determined with the Pierce® BCA Protein Assay Kit (Thermo Fisher Scientific, Wilmington, DE, USA) following the manufacturer’s instructions. Briefly, a series of dilutions of bovine serum albumin (BSA) were prepared as a set of protein standards to determine the standard curve. Then the BCA working reagent (WR) was prepared by mixing 50 parts of BCA Reagent A with 1 part of BCA Reagent B (50:1, Reagent A:B). 25 µL of HCC or BHT protein was pipetted in replicate into a microplate well and 200 µL of the WR was added to each well, and incubated at 37 °C for 30 min. The quality and quantity of the total isolated proteins were measured and assessed at 570 nm with the Benchmark microplate reader (Bio-Rad, Hercules, CA, USA).

### Electrospray ionization mass spectrometry (ESI–MS)

The concentrations of PL species were determined by an automated ESI–MS method. Briefly, 35 µl lipid extract and internal standard mixture were combined with solvents so that the ratio of chloroform/methanol/300 mM ammonium acetate in water was 300/665/35, and the final volume was 1.2 ml. The unfractionated lipid extract in an autosampler vial was introduced by continuous infusion into the ESI source on a triple quadrupole MS/MS (API 4000, Applied Biosystems, Foster City, CA), using an autosampler (LC Mini PAL, CTC Analytics AG, Zwingen, Switzerland) fitted with the required injection loop for the acquisition time and presented to the ESI needle at 30 µl/min. PLs were detected by 80 cycles of ESI–MS (MS1) scanning from 150 to 400 u. The source temperature (heated nebulizer) was 100 °C, the interface heater was on, − 4.5 kV was applied to the electrospray capillary, the curtain gas was set at 20 (arbitrary units), and the two ion source gases were set at 45 (arbitrary units). The background of each spectrum was subtracted, the data were smoothed, and peak areas integrated using a custom script and Applied Biosystems Analyst software. The data were isotopically deconvoluted, and the PLs were quantified in comparison to a 15:0 internal standard. Peaks corresponding to the target PLs in these spectra were identified and molar amounts calculated in comparison to the internal standards for PLs. Finally, the data were corrected for the fraction of the sample analyzed and normalized to the sample volume to produce data in the unit of nmol/mg wwt HCC or BHT tissues, nmol/mg protein, and percentage of each phospholipid species out of total phospholipids according to the number of nmol/mg wwt.

### Data analysis

Statistical analysis was carried out in SPSS software (IBM SPSS Statistics 24 software). The mean concentrations of PL parameters between the two groups were compared by independent samples *T* test. Correlation and/or regression between two series of numerical data was analyzed by bivariate correlation and/or linear regression in Graphpad prism 8 software. Chi-square test was used to compare changes in percentage between two groups. Cluster3 and Treeview software and programs were used to graph heatmap and hierarchical clustering analysis (HCA). The R software (R Core Team 2016 R, https://www.R-project.org) was applied in bioinformatics analyses. The generalized linear model (GLM) with binomial distribution was used to predict disease and control status based on lipid concentration by R function of GLM. The package of ROCR was used to estimate, sensitivity, specificity, recall, precision, *F*-measure, and area under curve (AUC). Information gain (InfoGain) was determined with simple logistics classification algorithm (a supervised attribute ranking method). The significant *p* value was set at 0.05 for all results.

## Results

### Quality control, PL profiles, data acquisition, and abundance

**Fig. 1:**
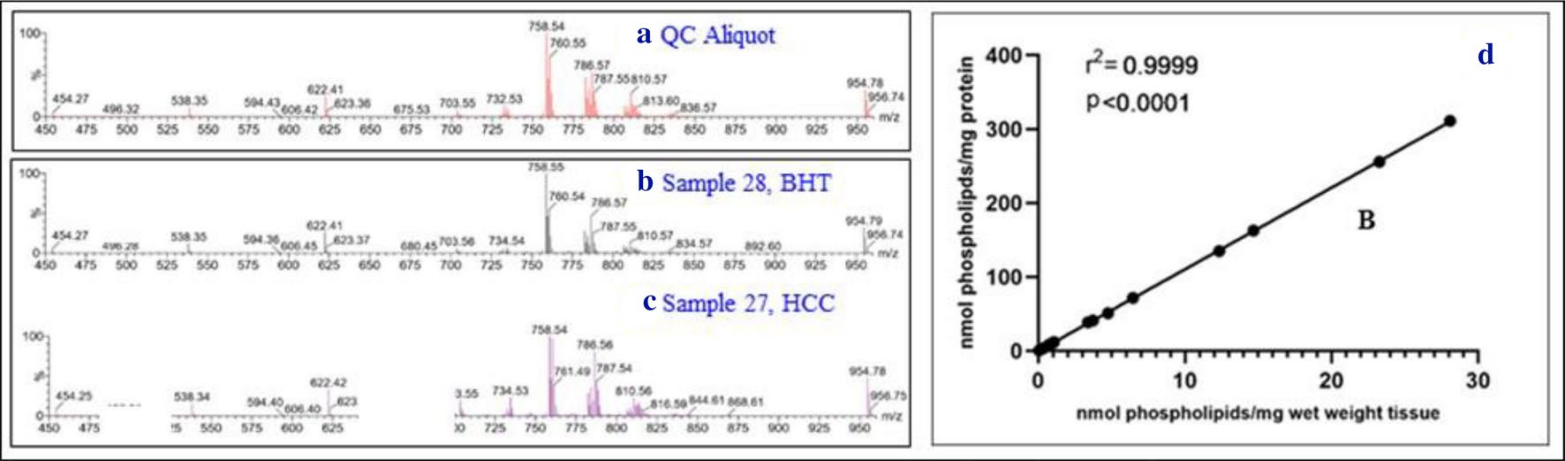
Representative mass spectra in ESI–MS analysis in the range of *m*/*z* 450–960 scanning mode for the detection of phospholipid species. **a** PL spectra represent lipid extract from one of ten aliquot quality control (QC); **b** PL spectra represent lipid extract from one of benign hepatic tissue (BHT) samples and **c** PL spectra represent lipid extract from one of hepatocellular carcinoma (HCC) samples; **d** correlation and regression analyses of mean concentrations in 13 PL classes between two quantitative methods. The plot showed a significantly linear correlation between mean concentrations of 13 PL classes in nmol/mg wet weight tissue (wwt) and that in nmol/mg protein (*r*^2^ = 0.9999, *p* < 0.0001)

To monitor the stability of the detection system and to minimize technical errors originating from sample collection and sample preparation [10], ten repeated aliquots of lipid extracts from pooled hepatic tissues were used as quality controls (QC). QC results demonstrated an acceptable reproducibility of metabolic features and a stability of ESI–MS profiling in 320 out of 335 PL species. The representative spectrums of QC, BHT, and HCC samples are shown in Fig. 1a–c, respectively.

To further prevent errors and to quantitate accurately and reliably, the concentration of each PL species in each sample was determined by two methods. One method was to calculate PL concentration in nmol/mg *wwt* fresh-frozen hepatic tissue. Another was in nmol/mg protein. The correlation and regression analyses were performed on the concentrations of total PLs in 13 classes between two methods. The results indicated that two methods are linearly correlated and this correlation was highly significant (*r*^2^ = 0.9999, *p* < 0.0001) as shown in Fig. 1d. In this study, the concentrations of PLs were represented by nmol/mg wwt tissues in data analysis. In addition, the percentage of given PL in individual species or class out of total PLs was used in the calculation of their abundances. Listed in Table 2 are the numbers of individual PL species in 13 PL classes and in six PL subgroups grouped according to similarity of moiety in phosphate head.

**Table 2:** PL profiles in hepatic tissues

Phospholipids (PL)	Number of PL species
Class	
Ceramide phosphorylethanolamine (PE-cer)	5
Dihydrosphingomyelin (DSM)	4
Ether-linked phosphatidylcholine (ePC)	25
Ether-linked phosphatidylethanolamine (ePE)	27
Ether-linked phosphatidylserine (ePS)	16
Lysophosphatidylcholine (LPC)	13
Lysophosphatidylethanolamine (LPE)	13
Phosphatidic acid (PA)	22
Phosphatidylcholine (PC)	46
Phosphatidylethanolamine (PE)	47
Phosphatidylinositol (PI)	46
Phosphatidylserine (PS)	48
Sphingomyelin (SM)	8
Total phospholipids	320
Subgroups	
Choline-containing PLs (ePC, LPC, PC)	74
Ethanolamine-containing PLs (PE-Cer, ePE, LPE, PE)	92
Ether-containing PLs (ePC, ePE, ePS)	68
Lyso-containing PLs (LPC, LPE)	26
Serine-containing PLs (ePS, PS)	64
Sphingomyelin-containing PLs (DSM, SM)	12

**Fig. 2:**
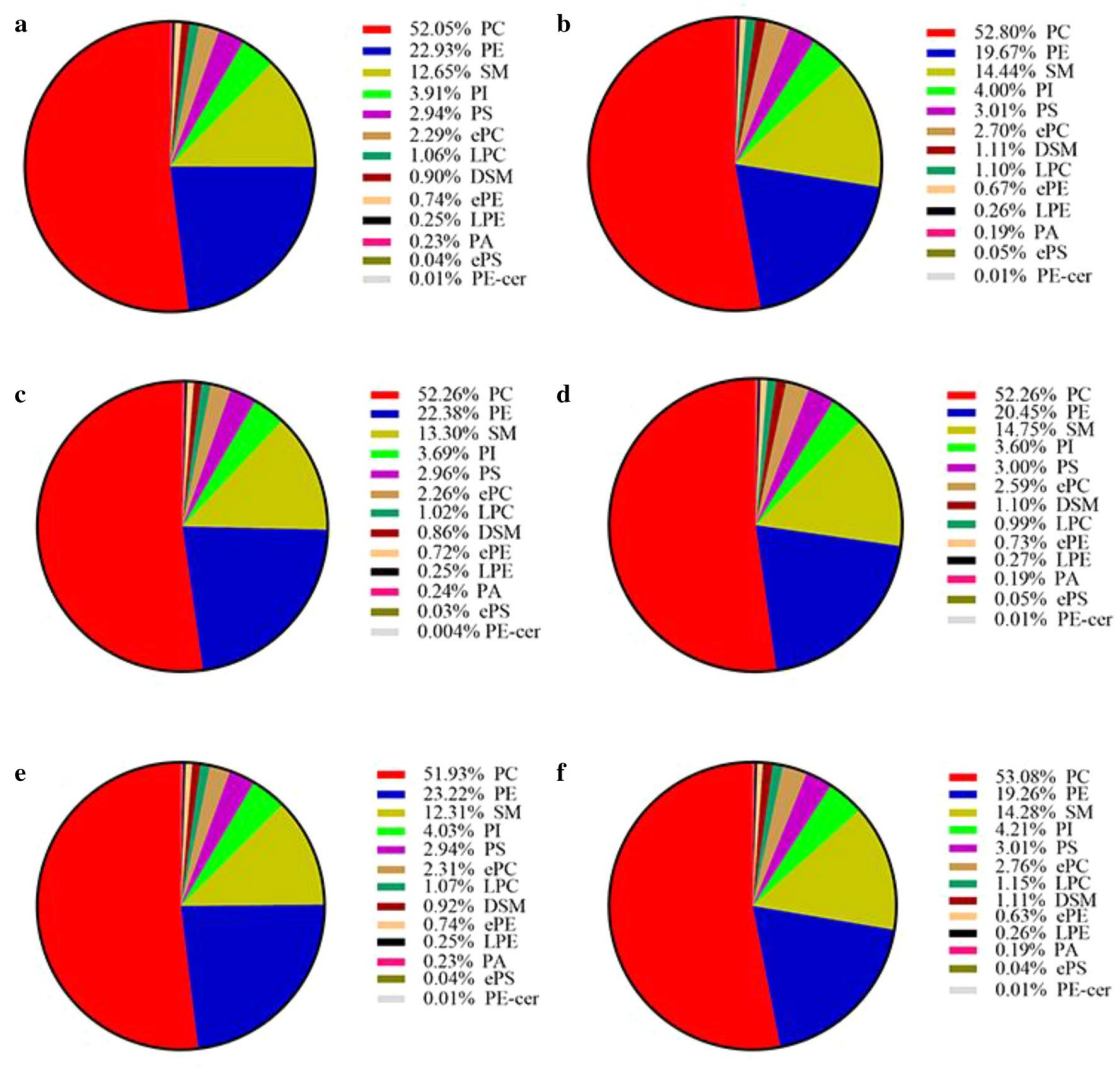
Abundances of 13 PL classes in BHT and HCC in all studied populations. **a** BHT and **b** HCC in all population; **c** BHT and **d** HCC in female population; and **e** BHT and **f** HCC in male population. PC was the most abundant PL class, and PE-Cer was the least abundant PL class in both BHT and HCC in all studied populations

The abundances of PLs varied greatly among individual species, classes and subgroups between BHT and HCC. In the level of individual PL species, PC 34:2 was the most abundant among 320 individual PL species and is lower in HCC than in BHT in all population (− 1.6-fold, *p* = 0.00010), female population (− 1.4-fold, *p* = 0.124), and male population (− 1.6-fold, *p* = 0.003). Top ten most abundant individual PL species account for more than 50% of total PLs in both BHT and HCC in all studied populations. In the level of PL class, among 13 PL classes, PC was the most abundant, accounting for more than 50% of total PLs, and PE-Cer was the least abundant, accounting for 0.01% or less of total PLs in both BHT and HCC in all studied populations (Fig. 2).In the level of subgroup, PE-containing PLs was the most abundant among six PL subgroups, accounting for more than 55% of total PLs in both BHT and HCC in all studied populations.

### Gender differences of PL concentrations in individual species between BHT and HCC

The differences of PL concentrations in 320 individual PL species, 13 PL classes, and 6 subgroups between BHT and HCC are listed in supplemental Tables 1, 2 and 3 for all population, stratified female population, and stratified male population, respectively.

**Table 3:** Differences of phospholipids in class, subgroup, and total between BHT and HCC among populations

Phospholipids in class/subgroup/total	All population		Female population		Male population	
	HCC/BHT ratio	*p *value	HCC/BHT ratio	*p* value	HCC/BHT ratio	*p* value
DSM	1.0	0.9421	1.1	0.6923	1.0	0.8285
ePC	0.9	0.2309	0.9	0.5192	0.9	0.3152
ePE	0.7	**0.0157**	0.8	0.4513	0.7	**0.0156**
ePS	0.9	0.5439	1.2	0.6500	0.8	0.1626
LPC	0.9	0.1927	0.8	0.4461	0.9	0.2965
LPE	0.9	0.4605	1.0	0.9718	0.9	0.3674
PA	0.7	0.0541	0.8	0.4782	0.7	0.0646
PC	0.8	0.1175	0.9	0.5516	0.8	0.1360
PE-Cer	1.5	0.3510	2.3	0.2497	1.2	0.6582
PE	0.7	**0.0149**	0.9	0.5726	0.7	**0.0073**
PI	0.9	0.4207	0.9	0.8014	0.9	0.4274
PS	0.8	0.1786	0.9	0.6498	0.8	0.1887
SM	0.9	0.3059	0.9	0.7045	0.9	0.3225
Choline-containing PLs	0.8	0.1176	0.9	0.5449	0.8	0.1379
Ethanolamine-containing PLs	0.7	**0.0151**	0.9	0.5713	0.7	**0.0076**
Ether-containing PLs	0.8	0.1087	0.9	0.5014	0.8	0.1394
Lysol-containing PLs	0.9	0.2198	0.9	0.5335	0.9	0.2924
Serine-containing PLs	0.8	0.1801	0.9	0.6622	0.8	0.1862
Sphingomyelin-containing PLs	0.9	0.3424	0.9	0.7466	0.9	0.3467
Total phospholipids	0.8	0.0813	0.9	0.5718	0.8	0.0808

The* p* value bolded are statistically significant

**Fig. 3:**
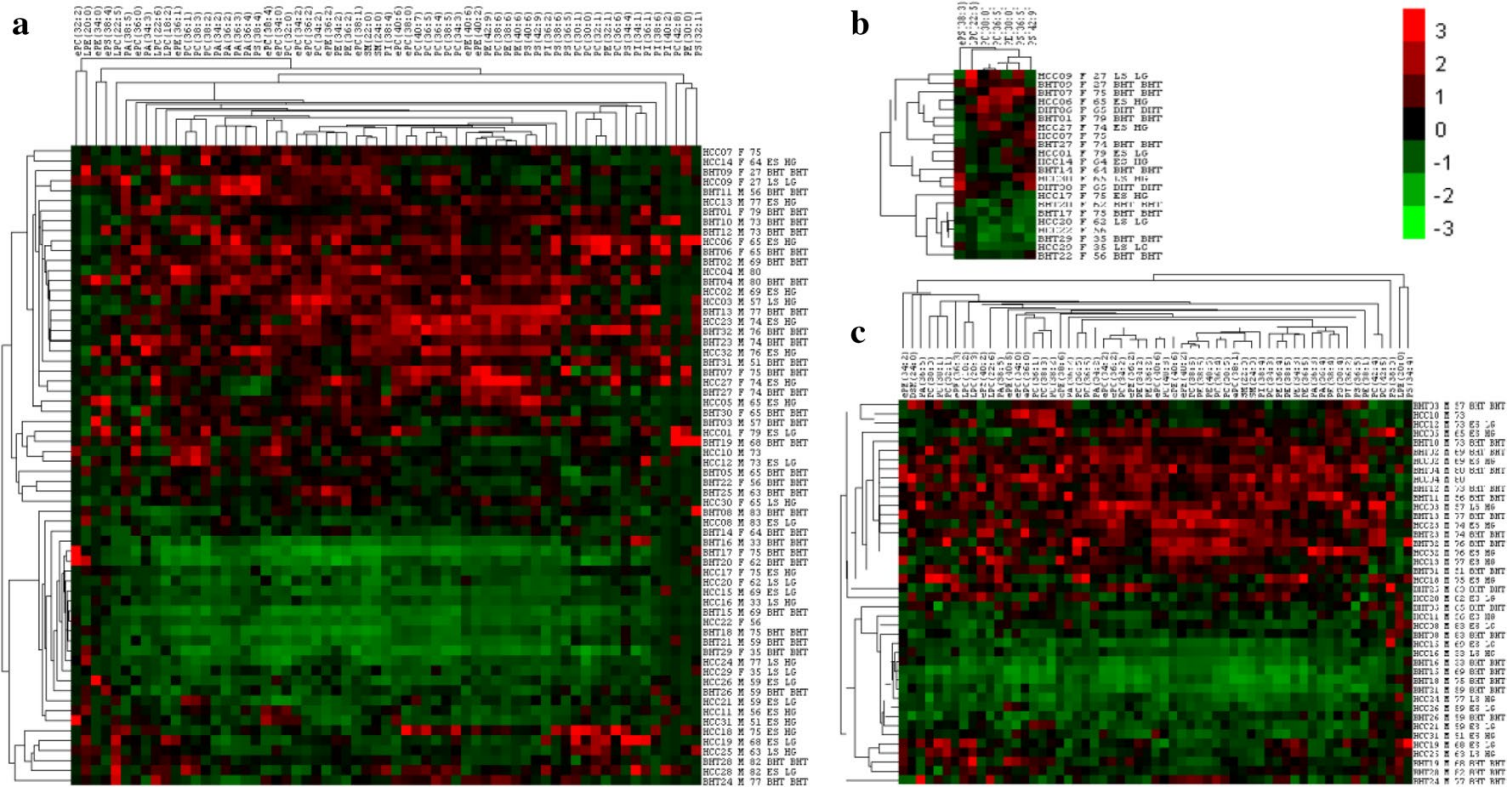
Heatmap and hierarchical cluster analysis (HCA) for individual PL species that was significantly different between BHT and HCC. The data were normalized by the *Z* score. The scale bars in color reflect changes in PL concentrations: squares in red represent higher PL concentration, and squares in green represent lower PL concentration. **a** heatmap and HCA for 62 individual PL species in all population; **b** heatmap and HCA for seven individual PL species in female population; **c** heatmap and HCA for 59 individual PL species in male population

In the level of individual PL species, 62 out of 320 PL species were significantly different between BHT and HCC in all population. Among them, 21 PL species were significantly increased, and 41 were statistically decreased in HCC than in BHT. The hierarchical cluster analysis (HCA) for the 62 species shown in Fig. 3a revealed that, the 62 individual PL species were scattered in 9 PL classes, among which PC had most (16) individual species, and the rest 8 PL classes had varied number of individual species ranging 1–9. The upper portion in Fig. 3a is predominated with individual PL species having higher concentrations, and the lower portion is predominated with individual PL species having lower concentrations. Interestingly, samples in upper portion have higher percentage of BHT (75% vs. 31%) and are more from patients with older age (67.9 vs. 61.9), lower percentage of females (29.6% vs. 39.9%), lower percentage of HCC in late stage (11% vs. 14%), and higher percentage of HCC in higher grade HCC (76.9% vs. 46.7%), as compared to samples in lower portion. Taken together, decrease in individual PL species is seen more in HCC than in BHT, and more related to younger male patients at late stage of HCC, but not to HCC grade in all population.

In female population, significant differences in concentrations between BHT and HCC were only seen in seven PL species: PC (30:0), ePS (38:3), LPC (22:5), ePC (34:0), ePE (34:0), PC (36:1), and PI (42:6) as shown in Fig. 3b. Six out of 7 PL species were significantly higher in HCC than in BHT (raged 1.7–20.6-fold), except LPC (22:5), which was 5.7-fold lower in HCC than in BHT. As well, the upper portion in Fig. 3b is predominated with higher concentrations of PL species, and lower portion in Fig. 3b is predominated with lower concentrations of PL species. However, it is difficult to correlate PL concentrations to the geographical and clinical features of samples, due to limited sample size.

In male population, significant differences in PL concentrations between BHT and HCC were seen in 59 out of 320 individual PL species. Unlike in female population, there were more PL species significantly lower in HCC than in BHT in male population: among 59 individual PL species, 11 PL species were significantly increased, and 48 were statistically decreased in HCC than in BHT. The hierarchical cluster analysis (HCA) for the 59 species as shown in Fig. 3c revealed that the 59 individual PL species were distributed in 12 of 13 PL classes, except for PL class of PE-Cers. Among 12 classes, PC class had most individual species (16) significantly different between BHT and HCC. The rest 11 classes had varied number of individual species ranging 1 to 10. Similarly, the upper portion in Fig. 3c is predominated with individual PL species having higher concentrations, and the lower portion in Fig. 3c is predominated with individual PL species having lower concentrations. The samples in upper portion have higher percentage of BHT (55% vs. 50%), and are more from patients with older age (69.8 vs. 63.9), lower percentage of HCC in late stage (11% vs. 30%), and higher percentage of HCC in higher grade (77.8 vs. 50%), as compared to samples in lower portion.

Taken together, in all population and male population, more PL individual species were significantly decreased in HCC than in BHT, and these decreased PL species more related to younger male HCC patients in late stage, but not to HCC grade. On the contrary, in female population, more PL individual species were significantly increased in HCC than in BHT.

### Gender differences of phospholipids in class, in subgroup, and in total between BHT and HCC

In the level of PL class, total ePE and total PE were significantly lower in HCC than in BHT in all population (*p* = 0.0157 and *p* = 0.0156, respectively), and stratified male population (*p* = 0.0149 and *p* = 0.0073, respectively). None of PL classes was statistically different between BHT and HCC in female population. While majority of PL classes were lower or unchanged in HCC than BHT, PE-Cer is the only PL class being obviously higher in HCC than in BHT in all studied populations, though the differences were not statistically significant.

PLs in all subgroups were lower in HCC than in BHT in all studied populations. However, significantly decreased PLs in HCC than in BHT was only seen in ethanolamine-containing subgroup in all population (*p* = 0.0157) and stratified male population (*p* = 0.0076), but not in stratified female population.

As well, the concentration of total phospholipids was lower in HCC than in BHT in all studied populations. As compared to female population, male population had about 10% more decreased in HCC than in BHT. All differences were not statistically significant. Table 3 showed differences of PLs in class, in subgroup, and in total between BHT and HCC.

### Identification of PL biomarkers in the diagnosis and prognosis of HCC

**Fig. 4:**
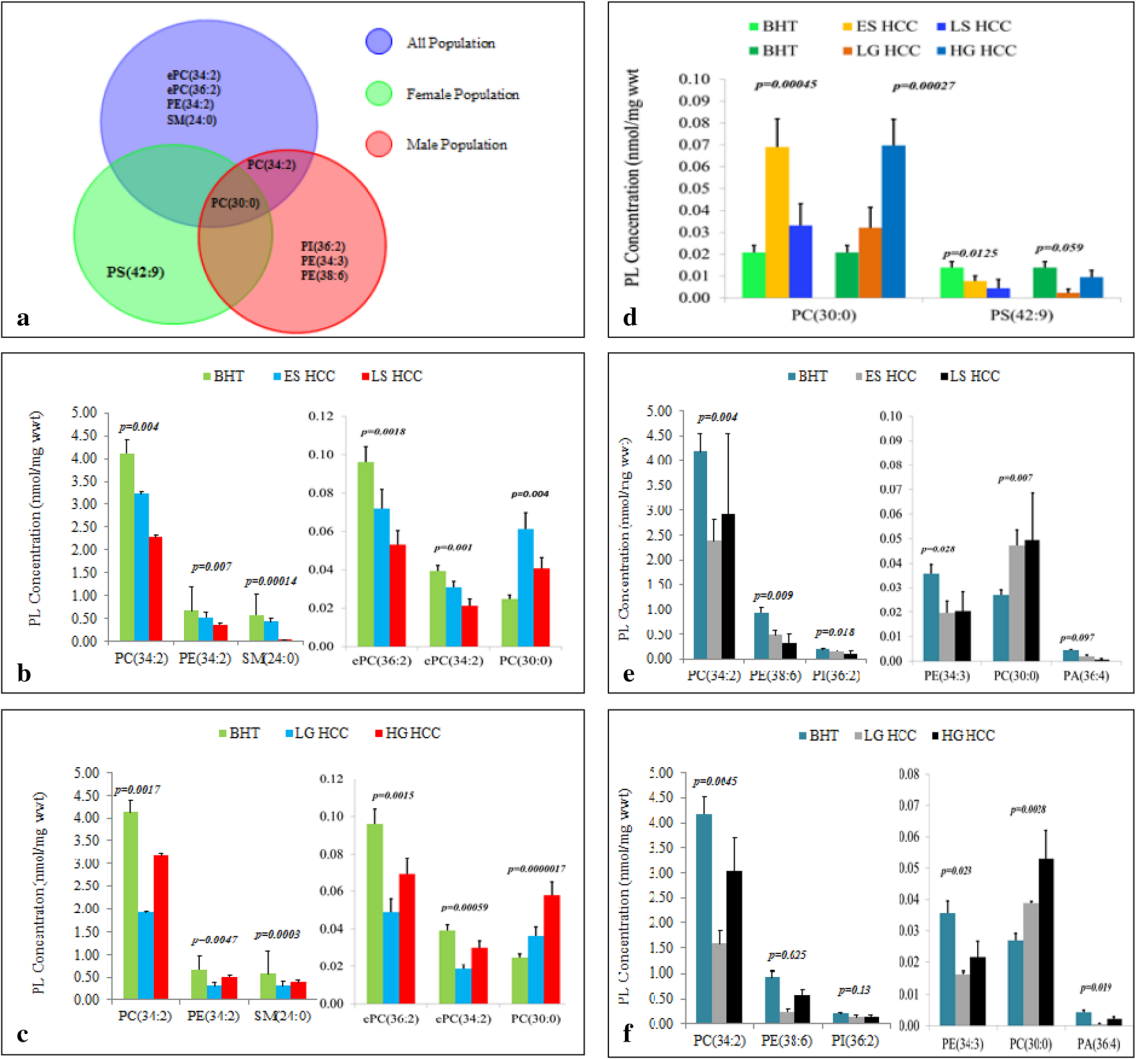
Distribution of PL biomarkers among populations and their correlation to clinical progression of HCC. *BHT* Benign hepatic tissues, *HCC* hepatocellular carcinoma, *ES* early stage, *LS* late stage, *LG* low grade, *HG* high grade. **a** The distribution of identified PL biomarkers in all studied populations; **b** correlation of identified PL biomarkers to clinical stage of HCC in all population; **c** correlation of identified PL biomarkers to pathological grade of HCC in all population; **d** correlation of identified PL biomarkers to clinical stage and pathological grade of HCC in female population; **e** correlation of identified PL biomarkers to clinical stage of HCC in male population; **f** correlation of identified PL biomarkers to pathological grade of HCC in male population. Data represent mean ± standard error and *p* < 0.05 was set statistical significance (ANOVA analysis)

Lipid profiling revealed that a number of PLs in individual species, classes, and subgroups were significantly different between BHT and HCC. To further identify if these PLs can serve as biomarkers in differentiation HCC from BHT, the following criteria were set for selection: (1) PL concentration was higher than detection limit (0.002 nmol/mg wwt) in at least one of BHT and HCC; (2) the differences in PL concentrations between BHT and HCC were statistically significant, no matter it is higher or lower in HCC; (3) all items of predicting power, especially sensitivity, specificity, and accuracy (AUC) were above 60% simultaneously. The PLs that fulfilled these criteria were all PL individual species, including six in all population: ePC (34:2), ePC (36:2), PC (30:0), PC (34:2), PE (34:2), and SM (24:0); two in female population: PC (30:0) and PS (42:9); six in male population: PA (36:4), PC (30:0), PC (34:2), PE (34:3), PE (38:6), and PI (36:2), as shown in Fig. 4a. Among these biomarkers, PC 30:0 is the only biomarker common to all studied populations, and PC (34:2) is a common biomarker to all population and stratified male population. Four biomarkers are specific to all population, one to female population, and four to male population.

To evaluate if these biomarkers are also able to predict the progression of HCC, these identified PL biomarkers in each study population were further correlated to clinical stage and pathological grade of HCC. ANOVA analysis indicated that all of the six identified PL biomarkers in all population significantly correlated to the progression of clinical stage (Fig. 4b) and pathological grade of HCC (Fig. 4c). In female population, only PC (30:0) did significantly correlate to the clinical stage and pathological grade of HCC (Fig. 4d). Except PI (36:2), the rest five PL biomarkers statistically correlated to the progression of clinical stage (Fig. 4e) and pathological grade (Fig. 4f) of HCC in male population.

Two of these identified biomarkers in PC class were unique. One was PC (30:0), which was the only identified PL biomarker that significantly higher in HCC than in BHT in all studied populations. Another one was PC (34:2), the most abundant PL species was lower in HCC than in BHT in all studied populations. Both increased PC (30:0) and decreased PC (34:2) in HCC were of strong diagnostic and prognostic powers for HCC, respectively. To test if the diagnostic and prognostic powers can be synergistically enhanced through the combination of these two PL biomarkers, the ratio of PC (34:2) to PC (30:0) was derived and evaluated in all studied populations. Analyses with methods in statistics and bioinformatics demonstrated that the ratio of PC (34:2) to PC (30:0) had much stronger power in the differentiation of HCC from BHT (Table 4) and in the prediction of the progression in context of clinical stage and pathological grade of HCC (Table 5), as compared to PC (34:2) or PC (30:0) alone.

**Table 4:** Diagnostic power of the ratio of PC (34:2) to PC (30:0) in all studied populations

Population		BHT	HCC	Sen	Spec	Prec	Recall	*F*-M	AUC
All	*n*	32	32	87.50%	93.75%	93.33%	87.50%	90.32%	94.43%
	Mean	1.66	0.65						
	SD	0.57	0.41						
	*p* value	0.000000000023							
	95% CI	0.76–1.26							
Female	*n*	11	11	81.82%	81.82%	81.82%	81.82%	81.82%	93.39%
	Mean	1.85	0.65						
	SD	0.69	0.39						
	*p* value	0.000067							
	95% CI	0.70–1.69							
Male	*n*	21	21	90.48%	95.24%	95.00%	90.48%	92.68%	95.24%
	Mean	1.56	0.65						
	SD	0.48	0.43						
	*p* value	0.00000011							
	95% CI	0.62–1.19							

*BHT* benign hepatic tissue, *HCC* hepatocellular carcinoma, *Sen* sensitivity, *Spec* specificity, *Prec* precision; *F-M* F measurement, *AUC* area under curve

**Table 5:** Prognostic power of the ratio of PC (34:2) to PC (30:0) in all studied populations

Population	BHT			ES HCC			LS HCC			*p* value*
	*n*	Mean	SD	*n*	Mean	SD	*n*	Mean	SD	
HCC clinical stage										
All	32	1.66	0.57	12	0.62	0.34	16	0.69	0.51	0.0000000028
Female	11	1.85	0.69	5	0.40	0.26	4	0.92	0.46	0.00057
Male	21	1.56	0.48	15	0.59	0.31	4	0.97	0.78	0.0000014
HCC pathological grade										
All	32	1.66	0.57	11	0.61	0.39		0.69	0.48	0.0000000028
Female	11	1.85	0.69	4	0.92	0.46	5	0.40	0.26	0.00055
Male	21	1.56	0.48	7	0.44	0.22	12	0.80	0.50	0.0000010045

*BHT* benign hepatic tissue, *HCC* hepatocellular carcinoma, *ES HCC* early-stage HCC, *LS HCC* late-stage HCC, *HG HCC* high-grade HCC

*p value was calculated by ANOVA

In summary, all identified PL biomarkers and, especially, derived ratio of PC (34:2) to PC (30:0) are not only able to differentiate HCC from BHT with high sensitivity, specificity, and accuracy but also are able to predict the progression of HCC in terms of clinical stage and pathological grade. As compared to female population, male population had more gender-specific PL biomarkers in the differentiation of HCC from BHT and in the prediction of HCC progression.

## Discussion

This study first employed ESI–MS techniques in determining global PL profiles in paired BHT and HCC samples from same female and male patients. We found that (1) the concentrations of PLs in total, in most subgroups and classes, and in majority of individual species were lower in HCC than in BHT; (2) such decreases of PLs in HCC were more prominent in male patients than in female patients; (3) a panel of individual PL species can be used as diagnostic and prognostic biomarkers for HCC, either common to all population, or specific to stratified female and male populations.

Phospholipids are essential components of all cells. Previous studies suggested PLs increase with cell transformation and tumor progression in many cancers such as breast cancer and colorectal cancer cells [11, 12]. None of the studies reported the differences in complete PL profiles between HCC and BHT samples. Few investigators performed partial PL profiles on plasma samples from HCC patients [6, 13, 14]. They found that several PL species were increased or decreased in plasma samples from HCC patients as compared to plasma samples from human subjects without cancers. Lu et al. also performed partial PL profiles on both hepatic tissues and sera simultaneously, and found that PL profiles in samples from sera and HCC tissues were not in agreement, implying that lipid metabolisms are likely to be modulated in different manners between HCC tissues and peripheral circulations [8]. Our results indicated that, unlike other cancers, HCC had lower concentrations of PLs in overall than that in BHT. This could be because of the facts that HCC cells are smaller in size than normal hepatocytes, and such reduction in HCC cell size becomes prominent in accordance with progressive thickening of tumor cell cords [15]. Actually, growth of HCC cells in smaller sizes are over-numbered HCC cells in larger size [16].

Each PL subgroup contains same head moiety. We found that the concentrations of PLs in all subgroups were lower in HCC. However, among PL subgroups, statistical difference was only seen in choline-containing subgroup between BHT and HCC in all population (*p* = 0.017) and stratified male population (*p* = 0.009). Phosphatidylcholines (PC) accounted > 93% choline-containing PLs (sphingomyelins were not included in this study) in both BHT and HCC in all studied populations. Decrease in PC and other choline-containing PLs in HCC might imply that the metabolism of these PLs favors degradation catalyzed by PC-specific phospholipase D in Kennedy’s pathway. On the other hand, the expression level of choline kinase-α, catalyzing the synthesis of phosphocholine is elevated in HCC [17] and other cancers. Thus, increase in degradation of choline-containing PLs and in the synthesis of phosphocholine might synergistically produce more phosphocholines in HCC tissues. Accumulated phosphocholines in HCC could serve as biomarkers in the diagnosis of HCC and in monitoring of therapeutic effects in the treatment of HCC by magnetic resonance spectroscopy imaging techniques, as demonstrated in HCC [18] and other cancer [19].

Ceramide phosphorylethanolamines (PE-Cer) are a group of sphingolipids that play critical roles in non-mammalian eukaryotes, similar to that of sphingomyelins in mammals [20]. Although it has been reported that mammalian cells also produce small amounts of the PE-Cers, the relationship of human PE-Cers with cancers have not been investigated. One study showed that PE-Cers isolated from *Sphingobacterium spiritivorum* ATCC 33861 can induce endonucleolytic DNA cleavage and apoptotic activity in human myeloid leukemia HL-60 cells in vitro [21]. PE-Cer was the least PL class, accounting for less than 0.01% in both BHT and HCC in all studied populations. Interestingly, PE-Cer was the only PL class with obvious higher concentration in HCC than in BHT in all studied populations. Further investigations are needed to clarify whether increase in PE-Cer produced in human HCC cells can also have similar functions in the induction of DNA damage and apoptosis against HCC cells seen in PE-Cers produced in bacteria on human myeloid leukemia HL-60 cells in vitro [21]. Perhaps it is a novel and potential therapeutic strategy to use exogenous PE-Cers in cancer treatment.

Effective therapies are feasible with surgical resection if HCC is diagnosed at an early stage [22]. However, the majority of patients are diagnosed at intermediate or advanced stage, resulting in high HCC-related deaths. Thus, identification of novel diagnostic and prognostic biomarkers is of great importance in early detection, monitoring therapy and predicting outcomes of HCC. To date, serum alpha-fetoprotein (AFP) remains the most commonly used biomarker in HCC. However, its use remains unsatisfactory and controversial, because AFP is only expressed in 60–80% of HCC cases [23] and because its sensitivity and specificity are inadequate [24]. Currently, a plethora of new diagnostic and prognostic biomarkers for HCC are in development, but have not been widely used in practice [25]. In our study, as compared to AFP, all identified PL biomarkers in hepatic tissues possessed higher sensitivity, specificity, and accuracy in the differentiation of HCC from BHT. However, these results were obtained from a limited size sample, and need to be further validated in a large study cohort.

Interestingly, PC (30:0) was found to be more abundant in breast cancer area as compared to the stroma surrounding the cancer, and considered as abnormal lipid metabolism-generated metabolites that promote cancer metastasis [26]. Kurabe et al. found that PC (34:2) was one of the few PC species highly downregulated in gastric cancer by imaging mass spectrometry (IMS) [27]. In agreement with these studies, we found that increased PC (30:0) and decreased PC (34:2) in HCC were able to differentiate HCC from BHT with high sensitivity, specificity, and accuracy simultaneously while they were used alone. When the ratio of PC (30:0) to PC (34:2) was used, the powers in differentiating HCC from BHT (Table 4) and in predicting clinical progression of HCC (Table 5) were dramatically amplified. Of course, these PL biomarkers need to be further validated in larger study cohorts as well.

The incidence, morbidity, and mortality rates of HCC are obviously disparate between male and female populations [28, 29]. The molecular mechanisms underlying these gender disparities have not been fully elucidated yet. Given that alterations in PLs between BHT and HCC correlate with occurrence, progression and clinical outcomes of HCC, gender differences in PL alterations should be in parallel reflected to these disparities. As compared to female HCC patients, male HCC patients had lower HCC to BHT ratio of total PLs (0.80 in male and 0.89 in female), more PL individual species significantly decreased in HCC than in BHT (59 vs.7), and more gender-specific PL biomarkers identified (4 vs. 1). Also, the identified male-specific PL biomarkers had relatively higher power in the differentiation of HCC from BHT, and in the prediction of clinical progression of HCC than female-specific PL biomarkers. In addition, male HCC patients had lower concentrations of PER-Cer in HCC than in BHT, although it is not sure yet that increasing in PE-Cer is of protective effect on the oncogenesis of HCC. Overall, differences in the alterations of PL in HCC between female and male are mirrored gender disparities in epidemiological and clinical manifestations of HCC.

Male gender, onset of HCC at younger age, late clinical stage, and higher tumor grade are all associated with poor prognoses of HCC. In agreement, our study indeed revealed that decreased hepatic PLs were more related to male sex, younger age at onset, and HCC in late stage, but not to HCC grade. This could be due to the fact that only 2 out of 32 cases were classified as Grade III, and the majority of cases were classified grade I and grade II, according to the Edmondson–Steiner Classification of HCC.

## Conclusions

In conclusion, the findings in this study could provide new evidence in understanding the roles of PLs in oncogenesis, progression and gender disparities, in exploring new PL biomarkers in diagnosis and prognosis, and in discovering novel therapeutic targets for HCC.

## Electronic supplementary material

Below is the link to the electronic supplementary material.

Supplementary file1 (XLSX 250 kb)
